# Correction: Suppan et al. Impact of Two Resuscitation Sequences on Alveolar Ventilation during the First Minute of Simulated Pediatric Cardiac Arrest: Randomized Cross-Over Trial. *Healthcare* 2022, *10*, 2451

**DOI:** 10.3390/healthcare11121799

**Published:** 2023-06-19

**Authors:** Laurent Suppan, Laurent Jampen, Johan N. Siebert, Samuel Zünd, Loric Stuby, Florian Ozainne

**Affiliations:** 1 Division of Emergency Medicine, Department of Anesthesiology, Clinical Pharmacology, Intensive Care and Emergency Medicine, Faculty of Medicine, University of Geneva, Geneva University Hospitals, 1211 Geneva, Switzerland; 2 ESAMB-École Supérieure de Soins Ambulanciers, College of Higher Education in Ambulance Care, 1231 Conches, Switzerland; 3 Department of Paediatric Emergency Medicine, Geneva Children’s Hospital, Geneva University Hospitals, 1211 Geneva, Switzerland; 4 Service de la Protection et de la Sécurité, 2000 Neuchâtel, Switzerland; 5 Genève TEAM Ambulances, 1201 Geneva, Switzerland

## 1. Text Correction

There was an error in the original publication [1]. We used a “manufacturer’s recommendation” regarding the compressions’ depth of ≥3 cm, which was actually erroneous since the manufacturer states that the compression depth should have been between 4 and 5 cm, instead of 3 to 5 cm (written ≥3 cm in the whole manuscript because there were no compressions above 5 cm).

Therefore, two minor modifications have been made:

A correction has been made to ***Section 2.2. Outcomes, last paragraph***:

“Because the pre-determined chest compression depth target was not reached during this study, an additional chest compression depth outcome considering an arbitrary target (≥3 cm) based on the mean compression’s depth rather than the guidelines was added post hoc.”

A correction has been made to ***Section 4. Discussion, paragraph number 3***:

“The cut-off used to determine whether chest compression depth was adequate was decided according to the manikin’s size. Since the initial analysis showed that compression depth was consistently shallower than expected, a supplementary secondary outcome was added post hoc using an arbitrary target of ≥3 cm (based on the mean compressions’ depth) to define adequate compression depth. No significant difference was found depending on the guidelines used, but the issue of manikin fidelity should nevertheless be considered. Indeed, high-fidelity simulations have been shown to improve compression quality [28], and manikins’ limitations should be clearly acknowledged by their manufacturers, who should strive to increase the fidelity of their simulation materials.”

## 2. Error in Table

In the original publication [1], there were two mistakes in Table 2. First, it appears that the value under the “Difference” column was wrongly pasted. While the difference for the proportion of compressions with correct chest recoil was indeed 6% [−8;20], the same values appeared for the CCF. However, the correct values for the CCF should have been −7% [−11;−2]. In addition, the outcome “According to the manufacturer’s target (≥3 cm)” should be renamed “According to the ≥3 cm target” for the reason explained above. The corrected Table 2 appears below. 

The authors state that the scientific conclusions are unaffected. This correction was approved by the Academic Editor. The original publication has also been updated.

## Figures and Tables

**Table 2:** Secondary outcomes, expressed as median [Q1;Q3].

Outcome	ERC Approach	AHA Approach	Difference
Number of ventilations	13 [12;15]	10 [8;10]	3.5 [3;5]
Ventilation’s volume	54 mL [37;61]	52 mL [43;63]	−1 mL [−6;3]
Proportions of ventilations			
- *Below target (<30 mL)*	4% [0;23]	0% [0;11]	0% [−2;7]
- *In target (30–70 mL)*	76% [65;82]	75% [52;100]	2% [−9;10]
- *Above target (>70 mL)*	3% [0;24]	0% [0;31]	0% [−3;0]
Alveolar ventilation with ventilation capped at 70 mL	365 mL [203;445]	271 mL [138;353]	78 mL [33;117]
Compressions’ depth	32 mm [28;34]	32 mm [30;35]	−1 mm [−2;1]
Proportions of compressions with adequate depth			
- *According to the manikin’s size (≥4.3 cm)*	0%	0%	0%
- *According to the ≥3 cm target*	91% [17;100]	89% [36;99]	0% [−15;3]
Compression rate	109 cpm [103;114]	110 cpm [104;114]	−1 cpm [−3;1]
Proportions of compression rate			
- *Below target (<100 cpm)*	0% [0;20]	3% [0;13]	0% [−2;2]
- *In-target (100–120 cpm)*	91% [57;98]	90% [55;96]	1% [−5;6]
- *Above target (>120 cpm)*	1% [0;7]	0% [0;9]	0% [−1;1]
CCF	57% [54;64]	66% [59;68]	−7% [−11;−2]
Proportion of compressions with adequate chest recoil	93% [42;100]	76% [34;92]	6% [−8;20]

AHA: American Heart Association; CCF: chest compression fraction; cpm: compressions per minute; ERC: European Resuscitation Council.
